# Chromosome-Level Genome Assembly Provides New Insights into Genome Evolution and Tuberous Root Formation of *Potentilla anserina*

**DOI:** 10.3390/genes12121993

**Published:** 2021-12-15

**Authors:** Xiaolong Gan, Shiming Li, Yuan Zong, Dong Cao, Yun Li, Ruijuan Liu, Shu Cheng, Baolong Liu, Huaigang Zhang

**Affiliations:** 1 University of Chinese Academy of Sciences, Beijing 100049, China; ganxiaolong@nwipb.cas.cn (X.G.); blliu@nwipb.cas.cn (B.L.); 2 Key Laboratory of Adaptation and Evolution of Plateau Biota, Northwest Institute of Plateau Biology, Chinese Academy of Sciences, Xining 810008, China; zongyuan@nwipb.cas.cn (Y.Z.); caodong@nwipb.cas.cn (D.C.); liyun@nwipb.cas.cn (Y.L.); rjliu@nwipb.cas.cn (R.L.); 3 Key Laboratory of Crop Molecular Breeding, Xining 810008, China; 4 BGI-Shenzhen, Shenzhen 518083, China; lishiming@genomics.cn (S.L.); chengshu@genomics.cn (S.C.); 5 BGI Institute of Applied Agriculture, BGI-Shenzhen, Shenzhen 518120, China

**Keywords:** *Potentilla anserina*, allopolyploids, genome assembly, gene family, sub-genome expression dominance, starch metabolism

## Abstract

*Potentilla anserina* is a perennial stoloniferous plant with edible tuberous roots in Rosaceae, served as important food and medicine sources for Tibetans in the Qinghai-Tibetan Plateau (QTP), China, over thousands of years. However, a lack of genome information hindered the genetic study. Here, we presented a chromosome-level genome assembly using single-molecule long-read sequencing, and the Hi-C technique. The assembled genome was 454.28 Mb, containing 14 chromosomes, with contig N50 of 2.14 Mb. A total of 46,495 protein-coding genes, 169.74 Mb repeat regions, and 31.76 Kb non-coding RNA were predicted. *P. anserina* diverged from *Potentilla micrantha* ∼28.52 million years ago (Mya). Furthermore, *P. anserina* underwent a recent tetraploidization ∼6.4 Mya. The species-specific genes were enriched in Starch and sucrose metabolism and Galactose metabolism pathways. We identified the sub-genome structures of *P. anserina*, with A sub-genome was larger than B sub-genome and closer to *P. micrantha* phylogenetically. Despite lacking significant genome-wide expression dominance, the A sub-genome had higher homoeologous gene expression in shoot apical meristem, flower and tuberous root. The resistance genes was contracted in *P. anserina* genome. Key genes involved in starch biosynthesis were expanded and highly expressed in tuberous roots, which probably drives the tuber formation. The genomics and transcriptomics data generated in this study advance our understanding of the genomic landscape of *P. anserina*, and will accelerate genetic studies and breeding programs.

## 1. Introduction

*P. anserina* (Poan) is a stoloniferous herb in Rosaceae which is widely distributed in the western regions of China, particularly in the Qinghai-Tibetan Plateau (QTP) [1]. It can produce different levels of stolon with roots and leaves at nodes during the growing season (Supplementary Figure S1). By virtue of fast clonal growth of the stolon and robust root system, Poan had become one of the dominant species in the alpine meadow. The aerial parts of Poan are considered as excellent forage for wild animals and livestock. The roots of Poan usually swell and form edible tubers in QTP [2]. It has been served as a tonic food and folk medicine for ages [3,4]. The tuberous root is rich in starch [4], amino acids [3], tannins [5], flavonoids [6], and triterpenes [7], and is a crucial plant-based food source for Tibetan people. Quite a few pharmacological studies have proved the medicinal functions of the extracts and constituents from Poan, including hepatoprotective [1], immunomodulatory [8], antitussive [4], and expectorant [4] effects. These research results confirmed that Poan possesses essential medicinal application value and has huge industrialization potential.

The Potentilla genus belongs to the Potentilleae tribe, Rosaceae family, including Anserina and other five major clades, which have been confirmed by both morphological and molecular data [9,10,11]. Up to now, over 300 species and numerous naturally occurring hybrids were recognized, showing an intricate relationship within Potentilla genus [11]. However, only one species in this genus was sequenced and assembled [12], with a contig-level genome assembly, which is far from enough to allow the phylogenetic study of such a diverse and important genus.

The lack of genomic information undoubtedly limited the molecular genetic study of Poan. Here, we reported a chromosome-level genome assembly of Poan (2n = 4x = 28), combining PacBio long reads, Illumina paired-end (PE) reads and high-resolution Hi-C data. Based on the reference genome, comparative genomics and phylogenetic analysis among Poan and its relatives was carried out. Moreover, transcriptomic analysis among different tissues were utilized for gene annotation and investigation of expression pattern of key genes involved in starch metabolism. All the data generated in this study will serve as a valuable resource for unraveling the molecular mechanism behind special traits of Poan and elucidating the evolution of Potentilla genus.

## 2. Materials and Methods

### 2.1. Plant Materials and High-Throughput Sequencing

Poan was collected in Guolog Tibetan Autonomous Prefecture, Qinghai province, China. The specimen used for sequencing were cultured in the greenhouse of the key laboratory of molecular breeding in the Northwest Institute of Plateau Biology, Chinese Academy of Sciences (CAS). High-quality genomic DNA was extracted from fresh young leaves using the Qiagen DNeasy Plant Mini Kit (Qiagen, Germantown, MD, USA). The quantity and quality of extracted DNA were examined with NanoDrop 2000 spectrophotometer (NanoDrop Technologies, Wilmington, DE, USA) and electrophoresis on 1% agarose gel, respectively.

Above all, PE reads with an insert size of 350 bp were generated using the NEB Next Ultra DNA Library Prep Kit according to the manufacturer’s instructions and further sequenced on Illumina HiSeq X Ten platform. For the long-reads library, 20 Kb SMRTbell libraries were constructed according to the BluePippin Size Selection System protocol as described by Pacific Biosciences (Pacific Biosciences, CA, USA) and then sequenced on PacBio sequel II platform with 5 SMRT cells. For Hi-C sequencing, one Hi-C library was constructed under the following procedure: chromatin was fixed with fresh formaldehyde to obtain the crosslink of DNA and protein. The crosslink was digested with Mbo I restriction endonuclease and 5’ overhangs were filled in with biotinylated nucleotides. After fixation, the adjacent fragments were ligated. Then, crosslinks were unlocked, and the DNA was extracted and sheared to a mean fragment size of 350 bp. Fragments with the biotin tag were captured using streptavidin beads and sequenced on the Illumina HiSeq X Ten platform.

In order to ensure the accuracy of gene annotation, the normal roots, tuberous roots, leaf, stem, shoot apical meristem (SAM), and flower of the Poan plant were collected for RNA-seq library construction. Then, all tissues mentioned above from another Poan plant were harvested for Iso-seq library construction. The RNA-seq libraries were generated using the NEB Next Ultra RNA Library Prep Kit for Illumina and sequenced on Illumina HiSeq 4000 to produce PE reads. The Iso-seq libraries were constructed and sequenced as the long-reads sequencing procedure mentioned above. To investigate the karyotype of Poan, we selected the stem apex tissue of Poan. The stem apex was treated with 0.1% colchicine for 4 h, then fixed for 20 to 24 h with Carnoy Fixative (acetic acid: absolute alcohol = 1:3). After that, the specimen was dissociated using 1 mol/L hydrochloric acid in water bath at 60 ${}^{{}^{\circ}}$C for 10 min. Finally, the specimen was dyed with nucleic acid dye (DAPI) for 10 min. The karyotype of Poan was scanned by a OLYMPUS BX63 automated fluorescence microscope under 100 × field of view.

### 2.2. Estimation of Genome Size and Genome Assembly

The qualified Illumina PE reads were used to estimate the genome size and heterozygosity through k-mer analysis. We adopted kmer_freq (https://github.com/fanagislab/kmerfreq (accessed on 10 May 2021)) and GCE (https://github.com/fanagislab/GCE (accessed on 10 May 2021)) for 17-mer analysis. The genome size was calculated according to the equation: genome size = k-mer_number/peak_depth. The ploidy of Poan was confirmed using Smudgeplot [13].

*De novo* genome assembling was performed using a FALCON (v0.2.2) assembler [14]. Firstly, the qualified PacBio subreads were self-corrected through a pre-assembled sequence. Then, overlaps were detected and filtrated for graph construction. Finally, contigs were generated using the graph. The initial contigs were aligned and polished with pbalign and Arrow programs as implemented in SMRT Link (v8) software (https://www.pacb.com/support/software-downloads/ (accessed on 8 April 2021)) with PacBio subreads. The refined contigs were further polished for two rounds using Pilon (v1.24) [15] (–fix snps, indels) based on Illumina PE reads, generating the draft genome assembly of Poan.

The qualified Hi-C reads were mapped to the draft genome assembly. We employed Hi-C pro (v3) [16] to obtain the valid pairs after duplication removal, sorting, and quality assessment. Subsequently, the valid Hi-C reads together with the draft genome assembly were processed following the 3D-DNA (20150322) pipeline [17] (-r 2 -i 2000). Finally, we obtained an explicit cross-linked pattern of sequences, which exhibited chromosomal arrangement. The cross-linked maps were visualized and manually checked using Juicebox (v1.11.08) [18]. To evaluate the assembled genome quality and continuity, we used three different methods. First, short PE reads were mapped back to the assembly genome using BWA [19] with the default parameters. Then, BUSCO (v5) [20] assessment was carried out (eudicots_odb10, –offline). Finally, LTR Assembly Index (LAI) [21] scores were calculated by LTR_Retriever (v2.8) [22] with the default parameters.

### 2.3. Non-Coding RNA and Repeat Identification

To identify the non-coding RNAs in the genome, tRNAscan-SE (v2.0.8) [23] and BLAST (v2.9.0+) [24] were used to detect the tNRA and rRNA, respectively. Meanwhile, the miRNA and snRNA were obtained using Infernal (v1.1.4) [25] based on the Rfam database [26]. The transposable element (TE) of genome were annotated in two ways including an *ab initio* method and homology-based search. RepeatModeler (v1.0.11) [27] and LTR_FINDER (v1.07) [28] with the default parameters were used for *ab initio* TE identification. For homology-based search, RepeatMasker (v4.1.0) (http://www.repeatmasker.org (accessed on 1 June 2021)) with default parameter was used to find the TE against the Repbase [29]. Furthermore, full-length LTR-RTs were collected for insertion time analysis using in-house Perl scripts.

### 2.4. Gene Prediction and Functional Annotation

Protein coding genes of the Poan genome were predicted combining three approaches, including *ab initio* prediction, homology-based alignment and transcriptome-based prediction. The gene prediction were performed on the basis of repeat-masked genome assembly. For *ab initio* prediction, we adopted AUGUSTUS (v3.3.3) [30] (–species = poan) and SNAP [31] (default parameter) to train and predict gene structure. For homology-based prediction, protein sequences from *Arabidopsis thaliana*, *Oryza sativa*, *Malus domestica*, and *Fragaria vesca* were downloaded from Phytozome database [32] and aligned to Poan genome sequences using tblastn [24] (expect (E) value cutoff: 1 × 10${}^{-5}$). Blast hits were filtered and integrated by in-house Perl scripts. Next, the sequences in Poan genome corresponding to targeted protein were extended upstream and downstream by 2000 bp to represent a protein-coding region. Finally, GeneWise (v2-4-1) [33] with -both -gff -quiet -silent -sum parameters was used to identify the exon and intron boundary. For transcriptome-based predictions, we build a comprehensive transcriptome database using genome-guided and *de novo* methods according to PASA pipeline (v2.4.1) with default parameter (https://github.com/PASApipeline/PASApipeline (accessed on 8 August 2021)). The Illumina PE reads were assembled under genome-guided mode using Trinity (v2.10.0) [34] and the PacBio long-reads were polished and clustered into full length transcripts through Iso-Seq (v3) pipeline with defult parameter (https://github.com/PacificBiosciences/IsoSeq/ (accessed on 8 August 2021)). At last, all evidences from three methods were integrated into a complete gene set without redundancy by EVidenceModeler (v1.1.1) with the default parameters [35].

Functional annotation of the predicted genes was actualized with BLASTP against various protein databases, including Pfam [36], Swiss-Prot (https://www.ebi.ac.uk/uniprot/ (accessed on 15 August 2021)), KEGG (https://www.genome.jp/kegg/ (accessed on 15 August 2021)), TrEMBL (https://www.ebi.ac.uk/uniprot/ (accessed on 15 August 2021)), and eggNOG [37].

### 2.5. Comparative Genomics and Phylogenetic Analysis

The nucleotide and amino acid sequences of seven Rosaceae species including: *Fragaria vesca* (Frve), *Malus domestica* (Mado), *Potentilla micrantha (Pomi)*, *Prunus persica* (Prpe), *Pyrus communis* (Pyco), *Rosa chinensis* (Roch), and *Rubus occidentalis* (Ruoc) were downloaded from the GDR database [38], while the sequence of *Arabidopsis thaliana* (Arth) was downloaded from the Phytozome database [32]. We used OrthoFinder (v2.5.4) [39] with default parameters to identify orthologous genes and gene family membership among nine species with default parameters. The species-specific gene families and expanded families were annotated and enriched using the topGO package (https://bioconductor.org/packages/release/bioc/html/topGO.html (accessed on 6 September 2021)) and KOBAS (v3) [40] based on the GO and KEGG databases.

For phylogenetic analysis, 286 single-copy genes were selected and aligned using MUSCLE (v3.8.31) with default parameters [41]. Subsequently, the aligned sequences were concatenated to generate a super-gene for each species using an in-house script. At last, we extracted the nucleotides at position 2 (phase 1) of each codon to construct phylogenetic tree using RAxML (v8.2.12 ) [42]. After maximum likelihood (ML) tree searching, one optimized ML tree were sequentially constructed with -m PROTGAMMAIJTTF and bootstrap value of 1000, with Arth as the outgroup. Furthermore, in order to investigate the speciation and divergence time of these species, we used the MCMCTree program with the default control file in PAML (v4.10.0) [43] to construct the time tree based on the ML tree. The calibrated timescales were obtained from TimeTree website [44]: Pyco–Mado (6–43 Mya), Arth–Mado (98–117 Mya). The gene synteny and gene duplication analysis was performed using the JCVI pipeline (https://github.com/tanghaibao/jcvi (accessed on 20 September 2021)).

### 2.6. Whole Genome Duplication (WGD) Analysis

To analyze the WGD event among Poan, Pomi and Frve, we characterized homologous genes using BLASTP (E-value cutoff: 1 × 10${}^{-5}$). Then by running MCScanX [45] with default parameters, we identified collinear blocks that included at least thirty collinear gene pairs. Finally, we calculated the Ks values of paralogous and orthologous gene pairs using PAML (v4.10.0) with default parameters, and depicted the distribution plot using R software. The WGD time was calculated according to the formula Time = Ks/2r (r = 7 × 10${}^{-9}$ mutations per site per year).

### 2.7. Sub-Genome Analysis and Expression Bias of Homeologs

To distinguish the genome structure of Poan, three different methods were combined. First, we investigated gene synteny within the Poan genome by JCVI, finding the existence of sub-genome structure. Then, we compared gene synteny between Pomi and Poan, confirming the order of sub-genomes. Finally, the homoeologous gene pairs (1:1) between sub-genome of Poan were obtained using Orthofinder. We further constructed a phylogenetic tree for each homoeologous gene pairs. The sub-genome with closer genetic distance to Pomi was denoted as the A sub-genome, and the remainder of the sub-genome was denoted as the B sub-genome. These gene pairs within the sub-genome were defined as homoeologous genes which were derived from latest WGD.

The homoeologous gene pairs were further used for expression analysis. We calculated the TPM value of homoeologs in tuberous root, normal root, stem, SAM, leaf, and flower. Log2(A/B) > 0 indicated the biased expression of A sub-genome homoeologs, and Log2(A/B) < 0 indicated the biased expression of B sub-genome homoeologs.

### 2.8. Identification of Resistance Genes and Starch Biosynthesis Related Genes

The conserved domains were collected from the Pfam database [36]. R genes were identified using HMMER (v3.3.1) [46] (E-value cutoff: 1 × 10${}^{-10}$). The protein sequences of key genes involved in starch biosynthesis were collected from the NCBI gene bank (https://www.ncbi.nlm.nih.gov/ (accessed on 12 October 2021)). All the target sequences were aligned with Poan, Pomi, Frve, Roch, Ruoc gene set using BLASTP (E-value cutoff: 1 × 10${}^{-10}$). After elimination of redundancy (identity less than 35% and coverage less than 60%), we confirmed the copy number of key genes in each species. For transcriptome analysis, low-quality reads and adaptors were filtered and trimmed using Trimmomatic (v0.39) [47]. Qualified reads were aligned to the genome assembly by HISAT2 (v2.1.0) [48]. Gene expression was measured as TPM using the featureCounts program (v2.0.1) [49] and R scripts. The expression level of key genes was displayed through pheatmap package (clustering_method = ‘ward.D’, scale = “row”).

## 3. Results

### 3.1. Genome Sequencing and Assembly

Young fresh leaves from the Poan plant were collected for *de novo* genome sequencing. To generate a high-quality genome assembly, we adopted hybrid methods including deep PacBio long-read sequencing, Illumina short-read sequencing, and Hi-C chromosome conformation capture. A total of 49.87 Gb qualified PacBio long-reads (∼108×), with reads N50 of 20.26 Kb, 44.45 Gb clean PE reads (∼96×), and 47.04 Gb clean Hi-C data (∼102×) were obtained (Supplementary Table S1).

**Figure 1:**
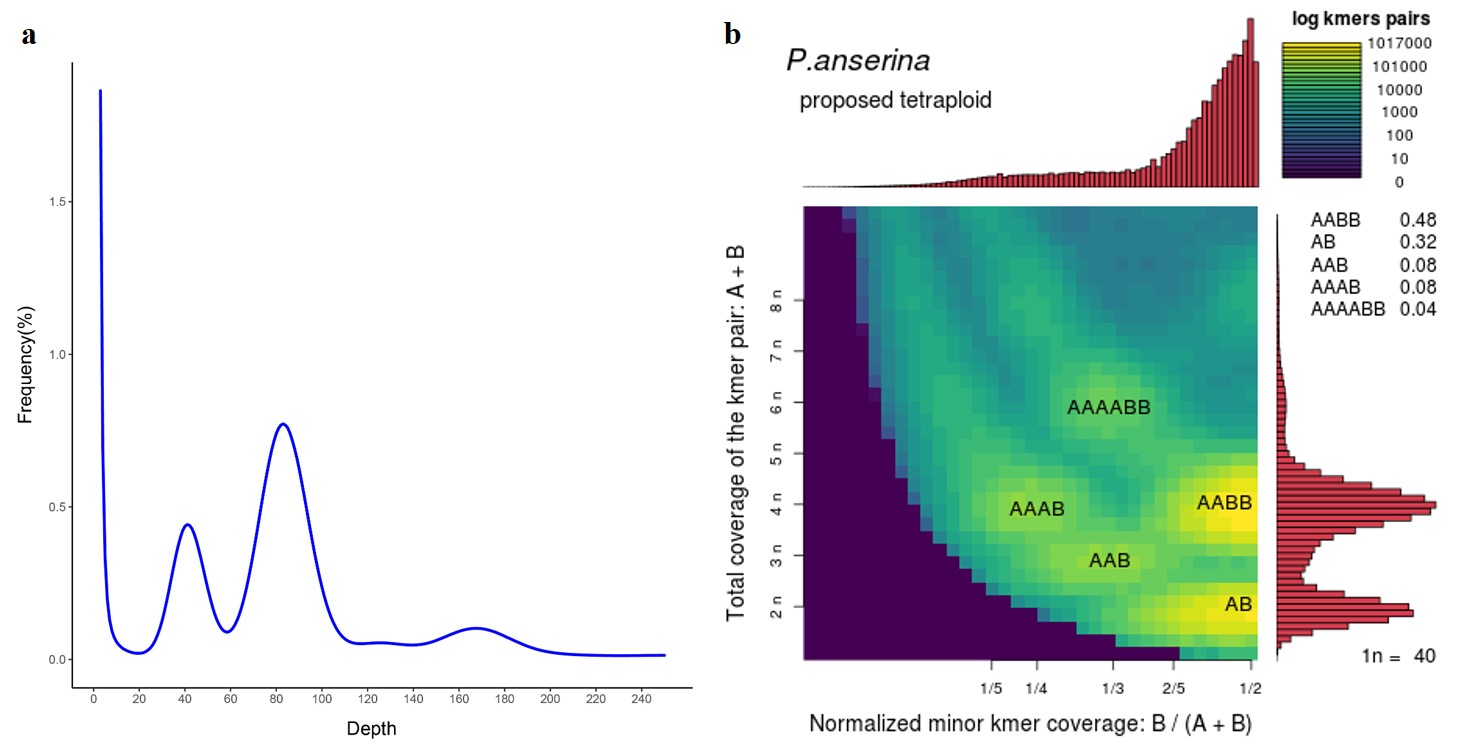
Genome size and ploidy analysis. (**a**) The k-mer (k = 17) frequency distribution. The sharp peak (∼83) is the homozygous region of genome and the smooth peak (∼168) reflected allotetraploidy. (**b**) The ploidy analysis for reference-free profiling of polyploid genomes.

We first investigated genome size using Illumina paired-end (PE) reads. The k-mer (k = 17) depth and frequency results showed that the Poan genome was 459.1 Mb with a heterozygosity rate of 0.7% (Supplementary Table S2). In addition, the sharp peak (82) in the k-mer frequency curve was expected from the homozygous region of genome, and the smooth peak (168) indicated the allotetraploidy of Poan [50,51] (Figure 1). The ploidy was further analyzed using the reported method for reference-free profiling of polyploid genomes [13], suggesting that the possibility of AABB was 0.48 (Figure 1), which confirmed the allotetraploidy of Poan. In addition, karyotype analysis showed that the sequenced Poan genome contained 28 chromosomes (Supplementary Figure S2). Considering the basic chromosome number in Rosaceae species was seven; therefore, Poan was an allotetraploid plant (2n = 4x = 28).

For genome assembling, all 49.93 Gb long-reads were self-corrected and assembled to a 487.44 Mb draft graph containing 1126 contigs, with a contig N50 value of 2.13 Mb. Eventually, the refined contigs were reordered and anchored into 14 chromosomes using Hi-C data (Supplementary Figure S3). The final assembly was 454.28 Mb, covering 98.95% of predicted genome size.

### 3.2. Assessment of Genome Quality

**Figure 2:**
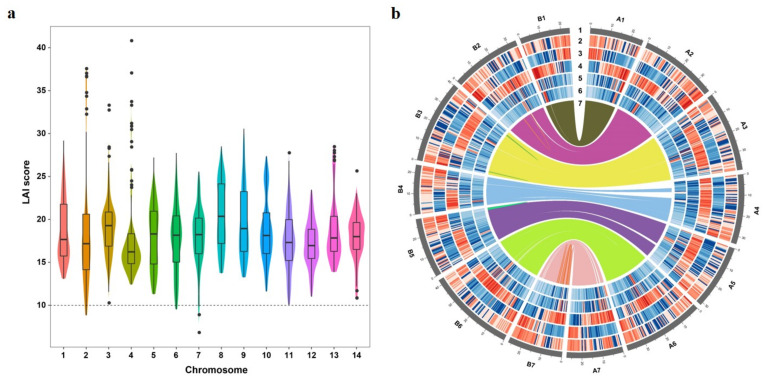
LAI scores and genome landscape of 14 chromosomes. (**a**) LAI scores distribution of Poan 14 chromosomes. The LAI score between 10 and 20 means the assembly reached the reference genome level. (**b**) Circos plot of genomic features. The tracks from the outermost to innermost are: 1 chromosome, 2 GC content, 3 gene density, 4 repeat density, 5 LTR-Gypsy density, 6 LTR-Copia density, and 7 collinear genes of Poan genome. The data are shown with a 500 Kb window.

In order to assess the quality of genome assembly, we adopted different methods. The Illumina PE reads were mapped back to the genome with a 99.49% mapping ratio, 94.39% of which was properly paired (Supplementary Table S3). We also counted coverage depth of each base and found that, of the mapped base pair, 98.86% had more than five depths. Then, BUSCO evaluation showed that 2288 (98.3%) completed BUSCOs and 7 (0.3%) fragmented BUSCOs were detected in the Poan genome (Supplementary Table S4), respectively, indicating a high completeness. Furthermore, LTR are the predominant type of transposable element (TE), which is poorly assembled in draft genomes. Ou et al. [21] proposed a metric called LTR Assembly Index (LAI) to evaluate assembly continuity. We therefore calculated the LAI scores of Poan 14 chromosomes. The average LAI score of Poan genome was 17.81, belonging to reference genome level. The highest median chromosome LAI score was 20.36 (chromosome 8), and the lowest median was 16.22 (chromosome 4) (Figure 2a). All these results implied the high integrity and continuity of the assembled Poan genome.

### 3.3. Genome Annotation

To obtain a high-quality gene set, we combined three approaches, including transcriptomic evidence, homology-based alignment and ab intio prediction. As a result, a total of 46,495 protein-coding genes were predicted. The average gene length, exon length, exon number per gene were 1963 bp, 230 bp and 5, respectively, which is similar to Pomi (Supplementary Table S5). The gene density was high in the distal region (Figure 2b). To obtain a better sense of functionality, 42,797 of the genes were successfully assigned to at least one public functional database. In detail, 92.05%, 82.05%, 46.53%, 79.36%, 39.32% of the protein-coding genes were assigned to Nr, Swiss-Prot, KEGG, TrEMBL, GO, respectively (Supplementary Figure S4). In addition, based on the Rfam database [26], 58.85 Kb, 83.08 Kb, 98.15 Kb, and 77.52 Kb of miRNA, tRNA, rRNA, and snRNA were identified in the Poan genome, respectively (Table 1).

TE has been considered to be the contributor of genetic variations in the plant genome [52]. Therefore, we used the *ab intio* method and homology-based method with Repbase [29] to annotate the repeats. Taken together, a total of 169.74 Mb (∼37.26%) TEs were identified (Figure 2b). The most abundant TE was LTR-RTs accounting for 23.2% (105.4 Mb) in total. Among LTR-RTs, *Gypsy* and *Copia* were two biggest TE sub-families, reaching 11.72% and 8.57% of the Poan genome, respectively. The DNA transposons, long interspersed nuclear elements, and short interspersed nuclear elements covered 9.50%, 2.82% and 0.06% of the genome, respectively (Table 1). The burst of LTR amplification usually affects the genome size and structure [53,54]. Therefore, we investigated the LTR insertion time of Poan, and two relatives (Pomi, and Frve). Interestingly, the LTR burst in Poan occurred∼0.18 Mya, which is slightly later than Frve (∼0.24 Mya) (Supplementary Figure S5). However, Pomi, another sequenced species in Potentilla genus, owned a totally different pattern of LTR-RT burst that happened∼1.36 Mya (Supplementary Figure S5), which was significantly earlier than Poan and Frve.

### 3.4. Comparative Genomics and Evolutionary Analysis

**Figure 3:**
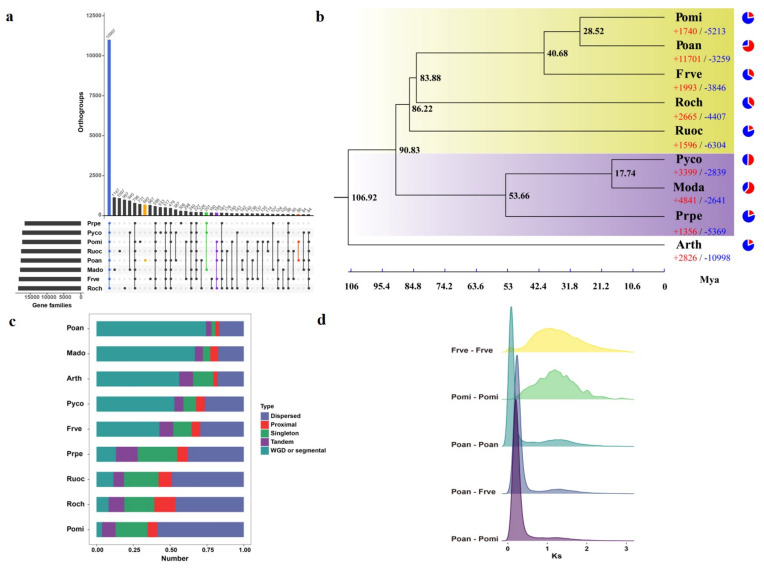
Evolution of the Poan genome. (**a**) Orthologous gene families among Rosaceae plants. (**b**) Phylogenetic relationship of Poan and eight other species. Herbaceous plants are shown with a purple background and woody plants are shown with a yellow background. (**c**) Gene duplication events among nine different species. (**d**) Ks distribution between Poan, Pomi and Frve.

Rosaceae is an economically important family with high diversity. The protein sequences of Poan and seven Rosaceae species, including Frve, Mado, Pomi, Prpe, Pyco, Roch, and Ruoc, were collected for comparative genomics analysis, with *Arabidopsis thaliana* (Arth) as the outgroup. Taken together, we found 29,221 gene families, of which 10,997 were shared by the Rosaceae species and 676 were specific to Poan (Figure 3a). GO enrichment analysis showed that the Poan-specific gene families were enriched in “response to light intensity”, “response to UV-B”, “lipid metabolic process” and other related biological processes (Supplementary Table S6). KEGG enrichment showed that the Poan-specific gene families were mainly enriched in “Biosynthesis of secondary metabolites”, “Galactose metabolism”, and “Starch and sucrose metabolism” pathways (Supplementary Table S7). We also identified 88 gene families which were specific to the Potentilla genus. In addition, a total of 201 and 185 gene families were shared by woody plants and herbaceous plants, respectively (Supplementary Table S8).

To investigate the evolution of the Poan genome, we selected 286 single copy genes to construct the phylogenetic tree. It is obvious that Pomi was the most closely relative of Poan. The Fragaria genus was close to the Potentilla genus. Poan, Pomi, Frve, Roch, and Ruoc were clustered into a clade that represented the herbaceous plants among the Rosaceae family, while Mado, Prpe, and Pyco represented the woody plants (Figure 3b). The herbaceous plants diverged from woody plants about ∼90.83 Mya (Figure 3b). The Potentilla genus separated from Fragaria genus about ∼40.68 Mya, followed by the divergence between Poan and Pomi (∼28.52 Mya). Based on the tree and multiple alignment sequences, we identified the expansion and contraction of gene families, which are crucial for environmental adaption of the plant. A total of 11,701 significantly expanded gene families (*p* < 0.05) and 3259 contracted gene families (*p* < 0.05) were found in the Poan genome. The expanded gene families were significantly enriched in 153 GO terms (biological process) including “response to hormone”, “hormone-mediated signaling pathway”, “reproductive shoot system development” and others (Supplementary Table S9).

Gene duplication plays a key role in novel functions and important traits of the plant genome [55,56]. We therefore investigated the gene duplication event among these species. Interestingly, the results showed that Poan possessed the most (858) WGD/segmental duplication involving 34,572 genes and the fewest (1279) singletons (Figure 3c).

The homologous gene pairs in Poan, Pomi and Frve were collected for whole genome duplication analysis. The synonymous substitutions per site (Ks) distance of these gene pairs revealed that Poan, Pomi and Frve shared a common ancient WGD (Ks∼1.05–1.2), while Poan underwent an additional WGD (Ks∼0.08), leading to the tetraploidization. The shared ancient WGD occurred at ∼70–80 Mya, while the tetraploidization of Poan occurred at ∼6.4 Mya, which was later than the divergence between Poan and Pomi, as well as Poan and Frve (Figure 3d).

### 3.5. Sub-Genome Structure and Expression Bias Analysis

**Figure 4:**
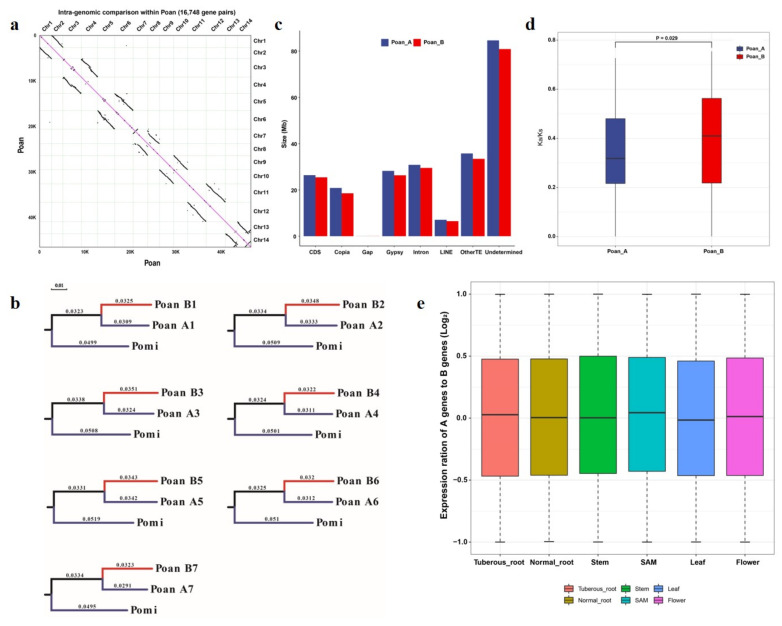
Sub-genome structure and expression dominance analysis. (**a**) Intra-genome gene synteny within Poan 14 chromosomes. (**b**) The phylogenetic tree of single copy genes between Pomi and Poan sub-genomes. (**c**) Sub-genome constitution of Poan. (**d**) Ka/Ks values of two sub-genomes. (**e**) Expression values for co-expression of homoeologous gene pairs.

Given that the Poan is an allotetraploid plant, it is necessary to dissect the sub-genome structure of the Poan. We adopted inter- and intra-species gene synteny and evolutionary analyses. First, we identified 16,748 gene pairs within the Poan genome, showing a high collinearity among 14 Poan chromosomes (Figure 4a). The 14 chromosomes were clearly divided into two groups, indicating the existence of the sub-genome structure (Figure 4a). Furthermore, there were 26,032 gene pairs between Poan and Pomi, 51% and 13% of which showed a 1:2 or 1:3 synteny pattern (Pomi:Poan) (Supplementary Figure S6). The top 10 contigs of Pomi also showed a high collinearity with two sub-genomes of Poan (Supplementary Figure S7).

In order to distinguish the two sub-genomes, 6345 single copy gene pairs between Pomi and Poan sub-genomes were collected for constructing phylogenetic trees. A group of seven chromosomes (1,3,5,7,9,11,13) who had a closer genetic relationship with Pomi genes were denoted as A1-7, and the remaining group was B1-7 (Figure 4b). The statistic of two sub-genomes showed that the A sub-genome was larger in size, gene number, and TEs, whereas it was similar in gap number and GC content (Figure 4c).

Allotetraploidy can coordinate two different genomes through genetic modifications to exhibit stronger adaptability and vigor [57]. To investigate the expression pattern and evolution of homoeologous genes, sequence alignment and RNA-seq analyses was carried out. Above all, a total of 7701 homoeologous gene pairs were identified. The non-synonymous substitution rate (Ka) and Ks of homoeologous gene pairs showed that the homoeologous genes in the A sub-genome had a significantly lower Ka/Ks ratio (mean Ka/Ks = 0.34) than the B sub-genome (mean Ka/Ks = 0.38) (Figure 4d), indicating that the A sub-genome might evolved differently to the B sub-genome. Then, we calculated the expression level of homoeologous genes in various tissues containing tuberous root, normal root, stem, SAM, leaf, and flower. In total, 43,112 (92.7%) genes were expressed (TPM > 1 in at least one tissue). The overall expression of homoeologous genes in the A sub-genome was slightly higher than in the B sub-genome (Supplementary Figure S8). Of the 7701 homoeologous gene pairs, 6490 (84.27%) pairs had expression differences greater than two-fold change in at least one tissue, including 3321 and 3169 homoeologs with higher expression in the A and B sub-genomes, respectively. Furthermore, 1134 and 1075 homoeologs of the A and B sub-genomes had higher expression values in all the six tissues, respectively. In addition, 97 and 117 homoeologs exclusively expressed in the A and B sub-genome, respectively. When the homologous gene pairs were both expressed, the median expression level of A homoeologs was similar to B homoeologs in normal root and stem, and was higher in SAM, flower, and tuberous root, but was lower in leaf (Figure 4e).

### 3.6. Resistance Gene Number Variation

QTP was characterized by dry climate, low temperature and strong ultraviolet, which was unfavorable for the plant pathogens and insect pests. Resistance genes containing nucleotide binding site (NBS) domains provide resistance to pathogens [58]. We therefore characterized the Resistance (R) genes in Poan and Frve. A total of 528 R genes were identified, including 1 *TIR-NBS*, 18 *CC-NBS*, 29 coiled-coil-NBS-LRR (*CNL*), 80 *NBS-LRR* genes, 66 *NBS*, 334 *LRR-RLK* genes. Based on the same parameter, the R gene number was 1321 in Frve and Pomi, which was over two times that of Poan and three times that of Pomi. There were 268 copies of *TIR-NBS-LRR* in the Frve genome, while it could not be detected in Poan. For *NBS-LRR*, there were 213, 80 and 0 copies in Frve, Poan and Pomi, respectively. In addition, the copy number of *TIR-CC-NBS-LRR* in Pomi (109) is far more than Frve (1) and Poan (0).

### 3.7. Genes Involved in Starch Biosynthesis

**Figure 5:**
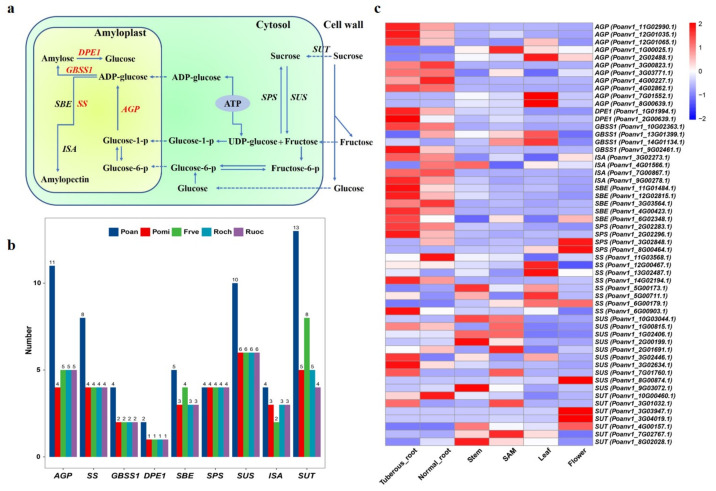
Analysis of key genes involved in starch metabolism. (**a**) A diagram depicting the main reactions involved starch metabolism. (**b**) Key gene copy numbers among Poan, Pomi, Frve, Roch, and Ruoc. (**c**) Heatmap of the expression level of genes related in starch metabolism in different tissues.

One of the most important trait of Poan is the tuberous root. Compared to the normal root, the tuberous root of Poan contains more starch. Therefore, we focused on key enzymes related to starch metabolism pathway. In root tissues, starch synthesis is derived from imported sucrose [59]. The sucrose was transformed into starch under a series of reactions [60,61]. The key enzymes involved in starch biosynthesis were expanded in Poan genome, including glucose-1-phosphate adenylyltransferase (*AGP*), the first key regulatory and rate-limiting enzyme in the starch biosynthesis pathway, starch synthetase (*SS*), granule-bound starch synthase1 (*GBSS1*), and disproportionating enzyme1 (*DPE1*). The gene numbers of *AGP*, *SS*, *GBSS1*, *DPE1* were 11, 8, 4, and 2, respectively, which is significantly more than those of Pomi, Frve, Roch, Ruoc (Figure 5a,b). Of the remaining starch biosynthesis-related genes, Poan still had the advantage of gene copy numbers (Figure 5b). Interestingly, the number of sucrose transporters (*SUT*) was increased to 13 in Poan (Figure 5b), four of which were tandem duplicated genes.

We further investigated the expression pattern of these key genes in different tissues. The results showed that seven *AGP* copies were highly expressed in tuberous root (Figure 5c), while 1 and 3 *AGP* copies were active in SAM and leaf, respectively (Figure 5c), indicating the gene subfunctionalization after allotetraploidization. Two *DPE1* genes had higher expression levels exclusively in tuberous root. *GBSS1* was highly expressed in both tuberous root and leaf (Figure 5c). Of other key genes, isoamylase (*ISA*), and the starch branching enzyme (*SBE*) were simultaneously exhibited up-regulated expression in tuberous root, while *SS* was expressed in leaf, stem and tuberous root (Figure 5c). Sucrose synthase (*SUS*) and *SUT* were quite active in different tissues (Supplementary Table S11).

## 4. Discussion

Poan is an important plant in QTP with immense economical and ecological value. In this study, we assembled a chromosome-level genome assembly and performed a series of investigation for uncovering the special features of Poan using genomic and transcriptomic information. The BUSCO assessment and LAI score results consistently indicated that the genome assembly of Poan showed a high contiguity and completeness, even in the repeat region. Our genome assembly is much better than Pomi assembly, another sequenced Potentilla species, whose genome assembly only reached contig level (contig N50 = 33.5 Kb) [12]. The Pomi genome has 2,674 contigs with 0.07% heterozygosity, which is quite homozygous compared to Poan genome [12]. In addition, the Poan genome size (454 Mb) was larger than Pomi (327 Mb) and had more genes due to the allotetraploidization [12]. The high-quality Poan genome assembly should be attributed to our massive sequencing data including PacBio long-reads, short PE reads and Hi-C data, and constantly revised assembling methods. The chromosome-level Poan genome should be the phylogenetic backbone of Potentilla genus and valuable resources for investigation of chromosome fusion and fission in Rosaceae family.

Based on the high-quality genome assembly, we investigated the evolution of Poan genome. The divergence between Poan and Pomi happened at ∼28.52 Mya. The WGD analysis suggested that, after the separation between them, Poan experienced the recent tetraploidization ∼6.4 Mya. The WGD introduced the gene family expansion and contraction. We found that the expanded gene families are involved in a lot of GO terms, some of which are related to the environmental adaption such as “respond to hormone” and “reproductive shoot system development”. The phytohormone biosynthesis and signaling may also play key roles in tuberous root development [62]. The rapid growth of stolons laid a solid foundation for reproduction and increase in tuberous root. The identified Poan-specific gene families are enriched in GO terms such as “response to light density” and “response to UV-B”. Light quality and intensity is one of the important factors of tuberous root formation [63]. It is important for Poan to adapt to the light density. Furthermore, Poan may have evolved the adaptability to survive under the strong ultraviolet light in QTP.

We adopted three methods including cytological evidence, k-mer analysis and ploidy analysis to investigate the ploidy of Poan. All evidences suggested that Poan was an allotetraploid plant. Through gene synteny and genetic distance analyses, we successfully identified the sub-genome structure of Poan. Each of them contained seven chromosomes, consistent with basic chromosomal number of Rosaceae species. The approaches used in this study provide a feasible strategy for investigating the allopolyploid plant genome.

Moreover, some studies had reported that Poan also has other varieties such as: pentaploid and hexaploid [64]. Therefore, our genome assembly of Poan represents the finite genetic variation of this species. More materials should be covered for identifying the more detailed genetic variations through re-sequencing or pan-genome assembling. However, based on our research, the typical features of Poan had been elucidated both in the genomic and transcriptomic aspects. The A sub-genome of Poan, genetically close to Pomi, was larger in size and evolved differently to the B sub-genome. The homoeologous genes had tissue-specific expression bias despite lacking significant genome-wide expression dominance. The overall R gene numbers in Poan and Pomi were obviously less than Frve, indicating the gene contraction in Poan and Pomi, which is probably be due to environmental adaption. The variation of R genes should lead to different disease resistance among these species. We further investigated the key genes involved in tannin, flavonoid and triterpene metabolism in Poan (Supplementary Table S12). Given that Poan are rich in these compounds, it is conducive to molecular research in the future. Furthermore, compared to other herbaceous plants, the key genes involved in starch biosynthesis were significantly expanded. It could be inferred that the copy number variation in the Poan genome was caused by tetraploidization. In addition, these key genes were highly expressed in the root tissue, and they evenly were distributed in the A and B sub-genomes (Supplementary Figure S9). These observed results strongly suggested that copy number variation caused by allotetraploidization and high expression level of key enzymes involved in starch biosynthesis lead to the tuberous root formation in Poan.

In conclusion, owing to the high-speed development of sequencing techniques, we assembled a reference genome of Poan, which shed light on the tuber formation and genome evolution. The data generated form this research will be significant resources for genetic studies and genomics-assisted breeding programs of Poan.

## Figures and Tables

**Table 1:** Statistics of Non-coding RNAs and TE sequences.

	Type	Number	Total Length (bp)	Proportion in the Genome (%)
	miRNA	479	58,847	0.013
	tRNA	1116	83,079	0.018
Non-coding RNA	rRNA	148	98,151	0.022
	snRNA	655	77,523	0.017
	Total	2398	317,600	0.07
	Retroelements	166,482	118,458,221	26.08
	LINEs	39,955	12,791,517	2.82
	SINEs	2373	269,946	0.06
	LTR elements	124,154	105,396,758	23.20
TE	*Gypsy*	56,850	53,227,095	11.72
	*Copia*	31,449	38,942,113	8.57
	DNA transposons	167,514	4,317,7431	9.50
	Others	27,886	6,454,876	1.42
	Total	368,838	169,737,457	37.26

## Data Availability

This Whole Genome Shotgun project has been deposited at GenBank under the accession PRJNA640225. All sequencing data are available at NCBI Sequence Read Archive SRP267963. The genome assembly and annotation files are available at figshare (https://figshare.com/projects/The_genome_assembly_and_annotation_files_of_Potentilla_anserina/83771 (accessed on 25 November 2021)).

## Supplementary Materials

The following are available online at https://www.mdpi.com/article/10.3390/genes12121993/s1, Figure S1: Morphological characteristics of *P. anserina*, Figure S2: Karyotype of Poan genome., Figure S3. The chromosomal contact matrix heatmap in Poan, Figure S4. Functional annotation of Poan gene set, Figure S5. LTR-RT insertion time of Poan, Pomi and Frve, Figure S6. The syntenic depth of all homologous gene pairs between Pomi and Poan genome, Figure S7. The syntenic blocks between top 10 contigs of Pomi and Poan genome, Figure S8. Overall homoeologous gene expression levels between A and B sub-genomes in Poan, Figure S9. Distribution of key genes involved in starch biosynthesis, Table S1. Summary of sequencing data, Table S2. Genome size analysis by k-mer (k = 17), Table S3. Illumina PE reads mapping result, Table S4. Statistics of completeness validation of genome assembly., Table S5. Summary of gene set of Poan and Pomi, Table S6. Significantly enriched GO terms of species-specific gene families, Table S7. Significantly enriched KEGG terms of species-specific gene families, Table S8. Orthogroups shared by woody plants and herbaceous plants, Table S9. Significantly enriched GO terms of expanded gene families, Table S10. Characterized R genes in F. vesca, *P. anserina* and *P. micrantha*, Table S11. The expression level (TPM) of key genes related to starch metabolism, Table S12. The key genes involved in tannin, flavonoid and triterpenes metabolism in Poan.
