# Supplementary material for: Chromosome-Level Genome Assembly Provides New Insights into Genome Evolution and Tuberous Root Formation of Potentilla anserina
Source: Genes (Basel). 2021 Dec 15;12(12):1993. doi: 10.3390/genes12121993 (PMC8700974; doi:10.3390/genes12121993)
Supplement: Supplementary file 1 [file genes-12-01993-s001.zip › Supplementary Figures.pdf]

genes

MDPI

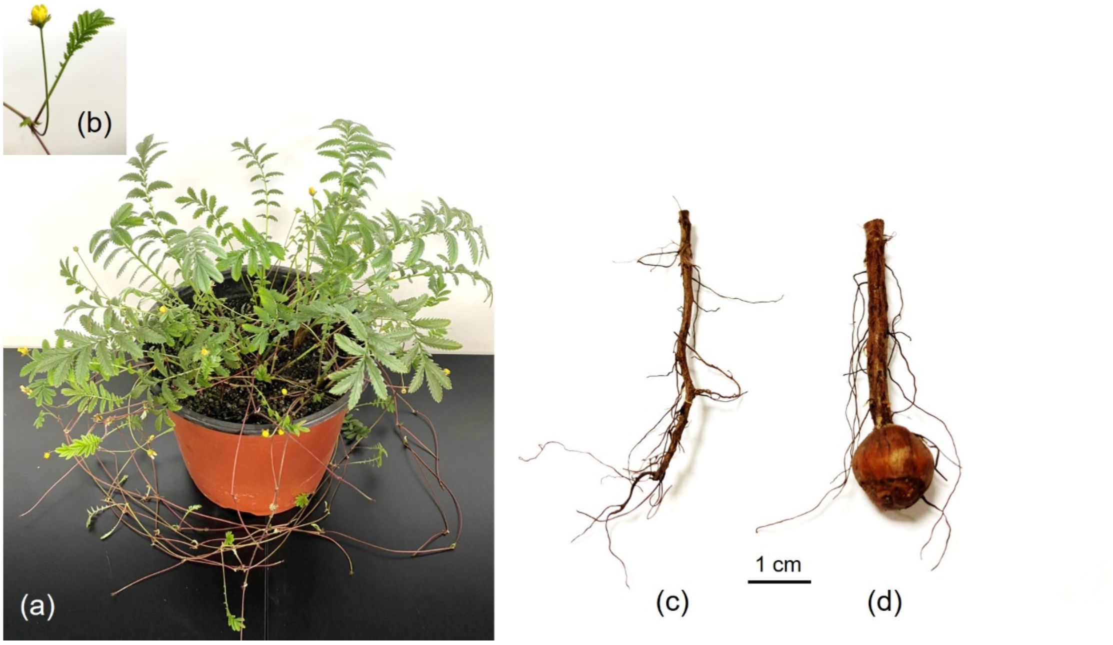

Supplementary Figure S1. Morphological characteristics of *P. anserina*. (a) Whole plant appearance. (b) Enlarged photo of internode with yellow flower. (c) Normal root. (d) Tuberous root.

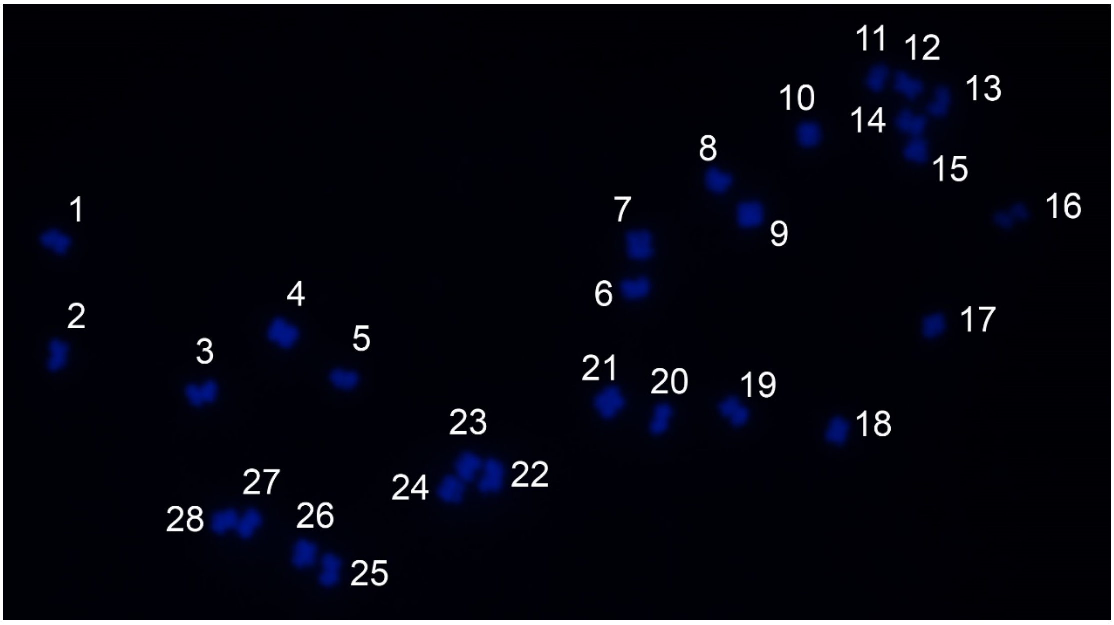

Supplementary Figure S2. Karyotype of Poan genome.

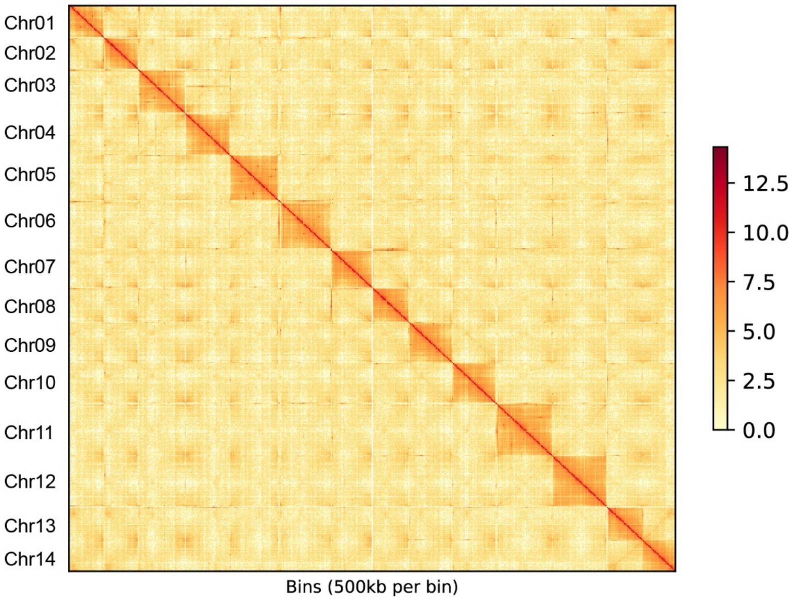

Supplementary Figure S3. The chromosomal contact matrix heatmap in Poan.

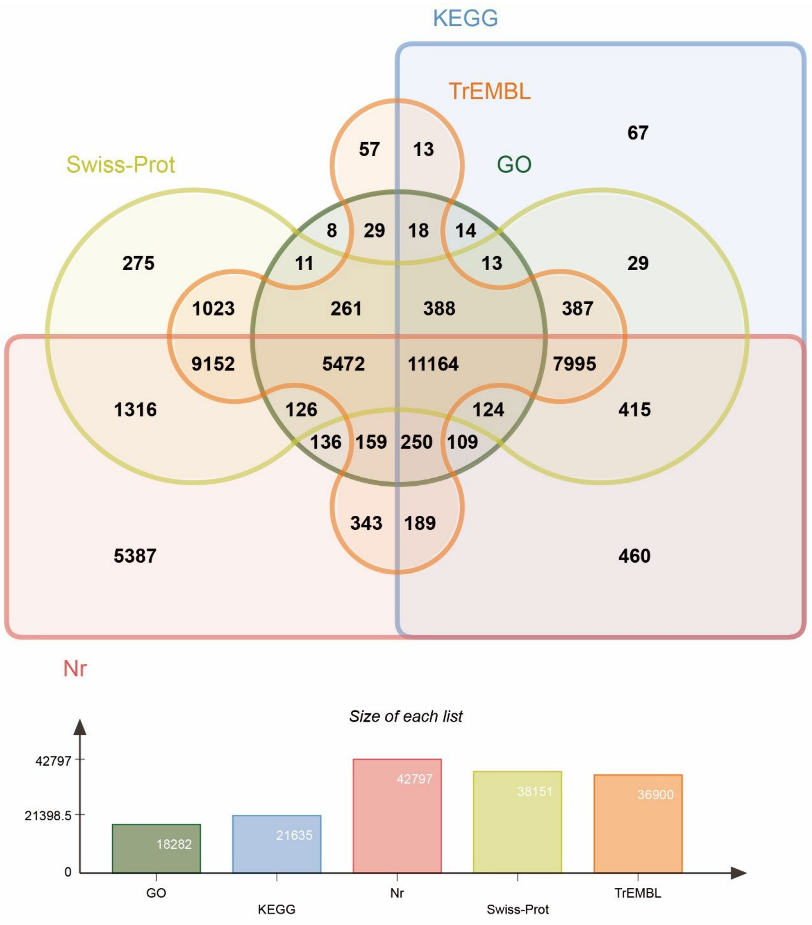

Supplementary Figure S4. Functional annotation of Poan gene set. A total of 92.05%, 82.05%, 46.53%, 79.36%, 39.32% of the protein-coding genes were assigned to Nr , Swiss-Prot, KEGG, TrEMBL, GO, respectively

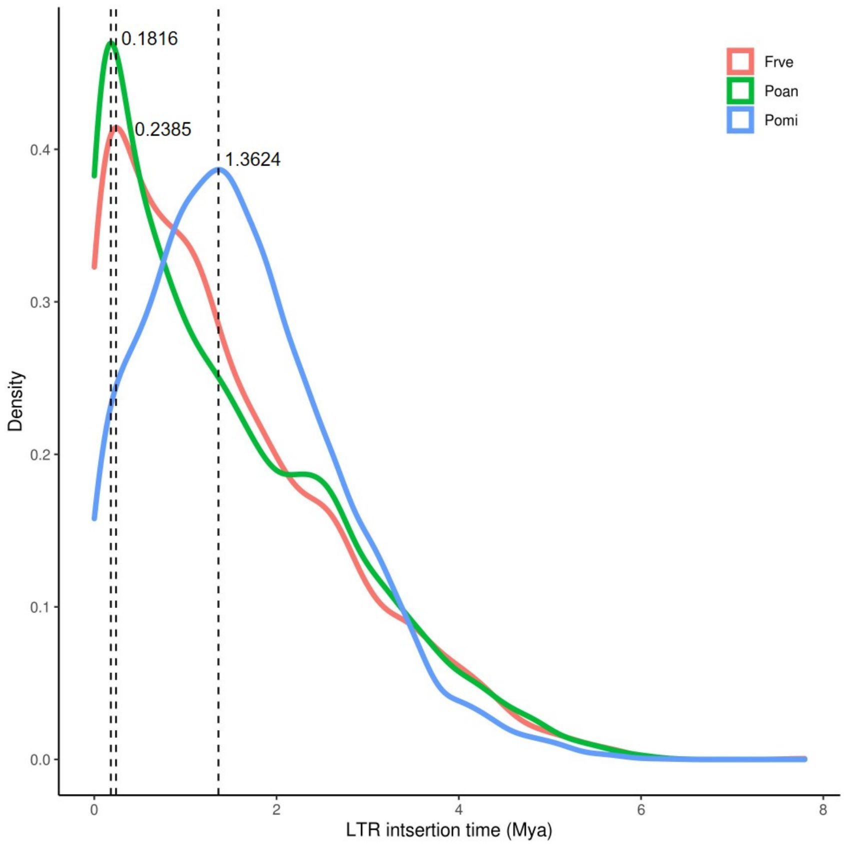

Supplementary Figure S5. LTR-RT insertion time of Poan, Pomi and Frve.

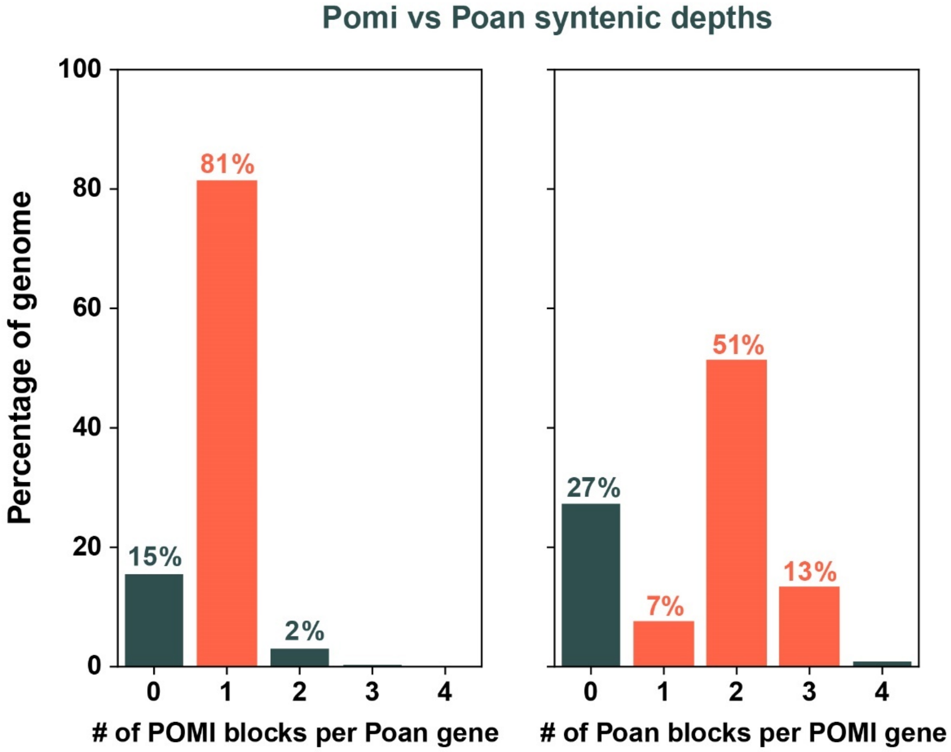

Supplementary Figure S6. The syntenic depth of all homologous gene pairs between Pomi and Poan genome.

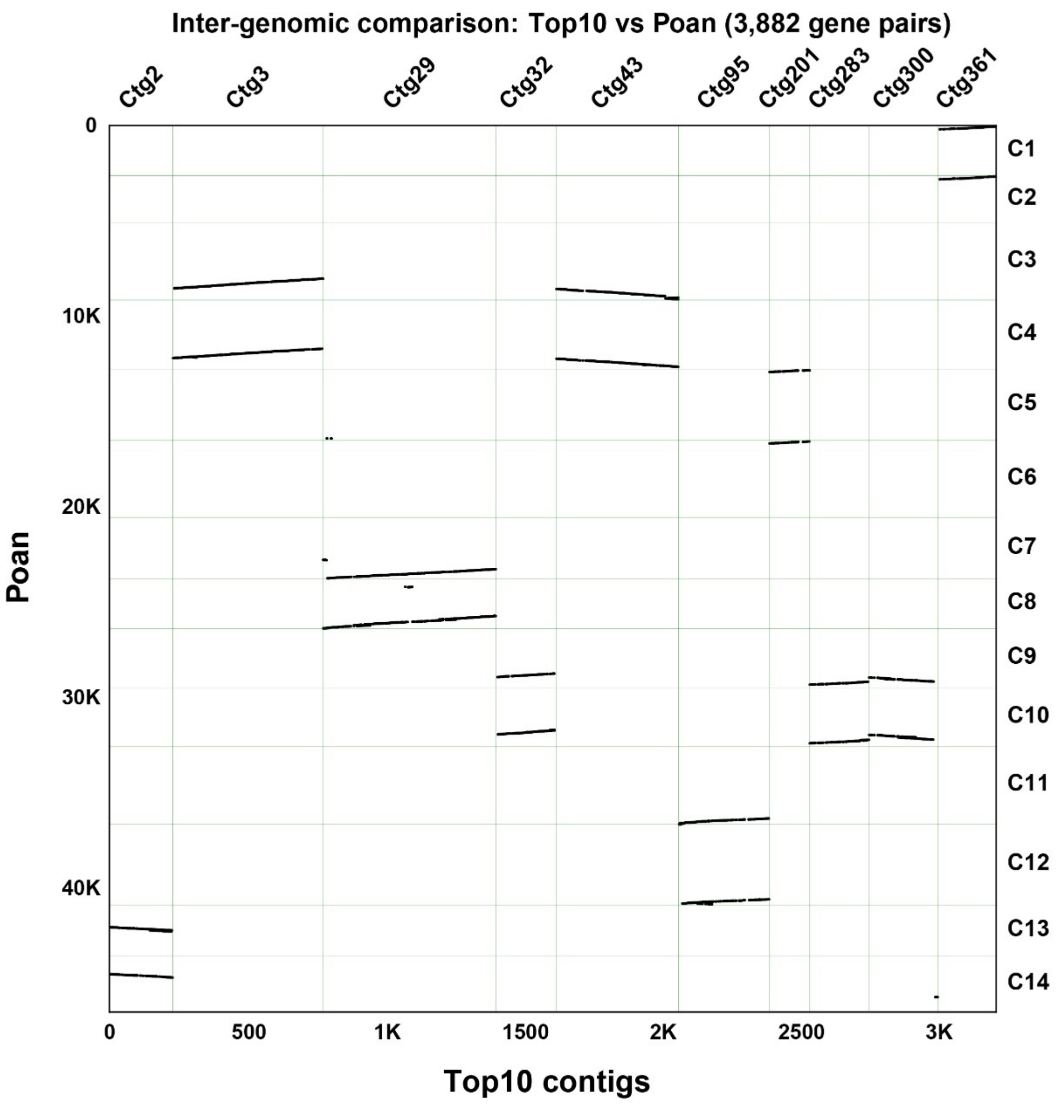

Supplementary Figure S7. The syntenic blocks between top 10 contigs of Pomi and Poan genome.

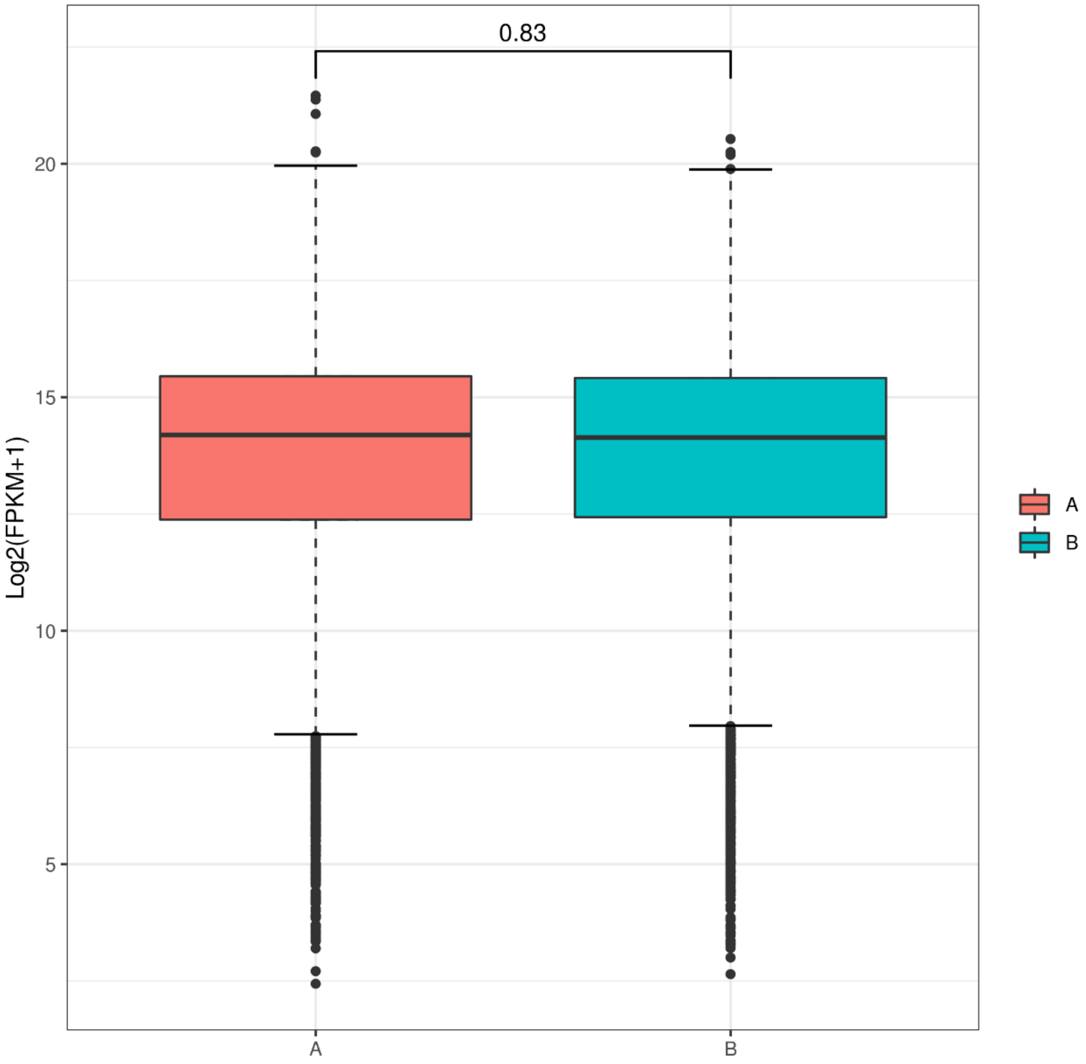

Supplementary Figure S8. Overall homoeologous gene expression levels between A and B sub-genomes in Poan.

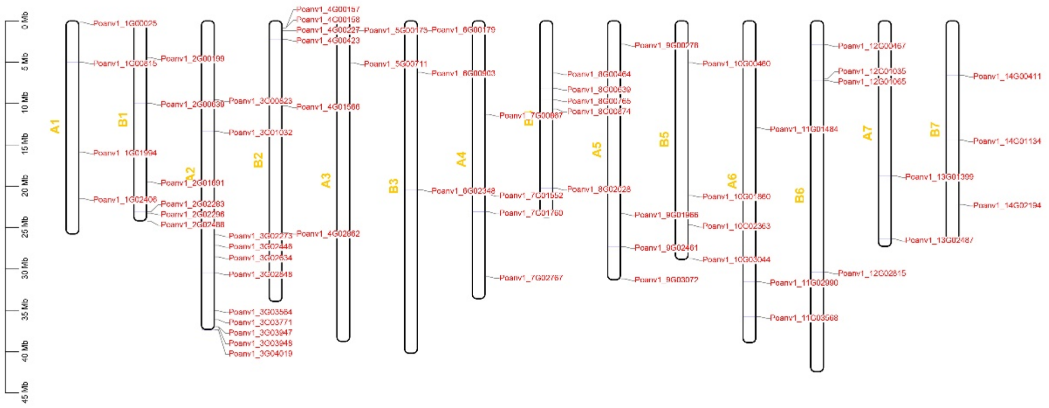

Supplementary Figure S9. Distribution of key genes involved in starch biosynthesis.
